# The Rapid TEG ***α***-Angle May Be a Sensitive Predictor of Transfusion in Moderately Injured Blunt Trauma Patients

**DOI:** 10.1100/2012/821794

**Published:** 2012-04-01

**Authors:** Victor Jeger, Sandra Willi, Tun Liu, Daniel D. Yeh, Marc De Moya, Heinz Zimmermann, Aristomenis K. Exadaktylos

**Affiliations:** ^1^Department of Emergency Medicine, University Hospital Inselspital Bern, 3010 Bern, Switzerland; ^2^Haemonetics Corporation, 400 Wood Rd., Braintree, MA 02184, USA; ^3^Trauma, Emergency Surgery and Surgical Critical Care, Massachusetts General Hospital, Boston, MA 02114, USA

## Abstract

*Background*. To guide the administration of blood products, coagulation screening of trauma patients should be fast and accurate. The purpose of this study was to identify the correlation between CCT and TEG in trauma, to determine which CCT or TEG parameter is most sensitive in predicting transfusion in trauma, and to define TEG cut-off points for trauma care. *Methods*. A six-month, prospective observational study of 76 adult patients with suspected multiple injuries was conducted at a Level 1 trauma centre of a university hospital. Physicians blinded to TEG results made the decision to transfuse based on clinical evaluation. *Results*. The study results showed that conventional coagulation tests correlate moderately with Rapid TEG parameters (*R*: 0.44–0.61). Kaolin and Rapid TEG were more sensitive than CCTs, and the Rapid TEG *α*-Angle was identified as the single parameter with the greatest sensitivity (84%) and validity (77%) at a cut-off of 74.7 degrees. When the Rapid TEG *α*-Angle was combined with heart rate >75 bpm, or haematocrit < 41%, sensitivity (84%, 88%) and specificity (75%, 73%) were improved. *Conclusion*. Cutoff points for transfusion can be determined with the Rapid TEG *α*-Angle and can provide better sensitivity than CCTs, but a larger study population is needed to reproduce this finding.

## 1. Introduction

The accurate and rapid detection of coagulation disorders and their treatment remains a challenge in emergency medicine. The development of acute traumatic coagulopathy (ATC) is related to several factors initiated by trauma and hypoperfusion. ATC is present in up to 25% of major trauma patients and is associated with a fourfold increase in mortality [1, 2]. While guidelines for blood product administration in ATC are evolving, laboratory analysis of the bleeding patient generally includes a panel of conventional coagulation tests (CCTs) that consists of the international normalised ratio (INR), activated prothrombin time (aPTT), thrombin time (TT), fibrinogen concentration, and platelet count [3]. However, CCTs have limitations. Firstly, INR, aPTT, and TT only measure one part of the coagulation cascade, the conversion of fibrinogen into fibrin. Other elements of coagulation are neglected [4]. Since aPTT and INR are performed with platelet-poor plasma, the contribution of platelets to clot strength is not measured. Secondly, CCT results may not be available during the crucial first 30 to 60 minutes following admission of the patient to the emergency department [5]. Some investigators believe that long waiting times can be addressed through implementation of point-of-care devices such as thrombelastography (TEG).

TEG provides a graphic representation of clot formation and lysis by assessing the viscoelastic properties of the clot over time [6]. TEG is a well-known point-of-care device, used primarily in liver and heart surgery [6]. It has been shown that the use of TEG in the operating theatre helps to reduce the use of blood products by at least 20% [7]. Although anaesthetists are familiar with TEG in the management of some surgical conditions, the device is not commonly used to monitor coagulation disorders at the bedside in trauma resuscitation. Kaufmann et al. [8] studied TEG in the trauma setting in the late 1990s, and work in this area has been continued by Tieu et al. [9] and Kashuk and Moore [10]. The introduction of TEG into trauma medicine has been supported by the development of Rapid TEG, in which tissue factor plus Kaolin are used to activate coagulation and which yields results within 20 minutes [11]. The main parameters are *R*: time from TEG start until initial fibrin formation; *K*: measure of time to reach a specified level of clot strength; *α*-Angle: rate of clot formation; MA: maximum amplitude. It has been reported that the TEG parameters correlate with blood product use and that TEG provides results more rapidly than conventional coagulation tests [9–12]. Rapid TEG parameters correlate with CCT in the trauma setting [11, 13]. Since the *R* and Delta (time to maximum rate of thrombus generation) parameters in TEG provide information on the enzymatic side of coagulation, they may correlate with PTT or INR.

The aim of this prospective observational study was to examine the correlation between conventional coagulation tests and TEG (both Rapid TEG and Kaolin TEG) in trauma, to determine which parameter is most sensitive in predicting transfusion in trauma and to define TEG cut-off points for optimal trauma resuscitation. 

## 2. Methods

A six-month prospective, nonconsecutive, observational study was conducted at Bern University Hospital (the Inselspital), a Level 1 trauma centre, where about 350 patients with injury severity score (ISS) >15 are treated per year. Patients were included if they were older than 16 years and had suspected multiple injuries, and a physician with TEG experience was available. Rapid TEG (tissue factor activated), Kaolin TEG, and conventional coagulation tests (INR; aPTT, TT, fibrinogen, platelet count) were all performed from citrated whole blood, as this is more practicable for a direct comparison between the different tests than using uncitrated whole blood [13]. The blood samples were collected by a phlebotomy nurse within 10 minutes of the patient's arrival. CCTs were performed in the central lab. All TEGs were run in the resuscitation bay. Temperature was set to 37°C for all samples. Citrated whole blood samples for TEG assays were recalcified by adding CaCl_2_. TEG instruments were tested for quality control weekly using standardised samples provided by the manufacturer. These results were always within range during the whole study period. Kaolin TEG and Rapid TEG samples were run in parallel on the same instrument. Physicians were blinded to TEG results. The decision to transfuse was based on clinical evaluation and prior threshold (cutoff) values for CCT. Injury Severity Score (ISS) was calculated from the Abbreviated Injury Scale (AIS), version 2008, from the Association for the Advancement of Automotive Medicine (AAAM) [14]. ICU scores (Acute Physiology and Chronic Health Evaluation II; APACHE II) and Simplified Acute Physiology Scores (SAPS II) were assessed by ICU staff at the bedside. As several definitions of ATC are in common clinical use, coagulopathy was analysed in four different ways: INR > 1.2, INR > 1.5, ATC 1 (INR > 1.5, aPTT > 60 seconds or TT > 15 seconds), and ATC 2 (INR > 1.2, aPTT > 60 seconds or TT > 15 seconds). Informed consent was waived by the Ethics Commission of the University Hospital, due to the purely observational character of the study.

Patients receiving blood products within the first 24 hours were compared to patients not requiring transfusion. Median values of laboratory parameters and of the demographic data were compared using a nonparametric Kruskal-Wallis test (for continuous variables) and a Fisher's exact test (for dichotomised variables), both for unpaired data. Pearson correlation coefficients were determined between parameters. Receiver operating characteristic (ROC) curves were generated for each test parameter, in order to determine sensitivity, specificity, PPV (Positive Predictive Value), NPV (Negative Predictive Value), and validity (AUC > 70%), using the Microsoft Excel program. ROC curves were generated and the optimum cutoff values selected by optimising the sensitivity and then the specificity, with the aim of achieving sensitivity of approximately 70% or higher, without compromising specificity.

## 3. Results

From November 2009 to May 2010, 85 patients met the inclusion criteria. This corresponds to about 50% of all multiply injured trauma patients in a 6-month period. Nine patients (11%) were excluded due to technical problems and handling errors, resulting in 76 patients for the final data analysis. The mean age of the overall population was 49 years and 72% were male. The injuries were predominantly blunt (83%); 43% of patients had suffered a craniocerebral injury. Mean ISS, APACHE II, and SAPS II were all consistent with moderate-to-severe injury and decreased probability of survival (Table 1).

Twenty-five patients (33%) received blood products during the first 24 hours after admission. Transfused patients were characterised by higher admission heart rates, more severe injuries, were more likely to be admitted to the ICU, and had higher overall mortality (Table 1). The median (range) of the number of administrations of blood products to transfused patients was as follows: RBCs, 4 (0–12); FFP, 2 (0–17) and platelet transfusions: 0 (0–3). Three patients in the transfused group did not receive RBCs; one was given FFP only and two patients had platelet transfusion only. Two of these three patients had a craniocerebral injury. The majority of patients who had an RBC transfusion were also given FFP (59%); five patients were only given RBC transfusions. Massive transfusion, defined as ≥10 units of RBCs in the first 24 hours after admission, occurred in 5 patients. These five patients were all given plasma transfusions that maintained RBC : FFP ratios at ≥0.7 (data not shown).

Blood samples on admission were analysed by CCT, Kaolin TEG, and Rapid TEG. When the four definitions according to conventional coagulation tests of acute traumatic coagulopathy were used, both INR > 1.2 and INR > 1.5 differentiated between transfused and nontransfused patients (Table 2). The CCT results illustrated that INR, aPTT, and fibrinogen were significantly different in transfused and nontransfused patients, as were the Kaolin TEG parameters K, *α*-Angle, MA, and G and the Rapid TEG parameters K, *α*-Angle, MA, TMA and G (Table 3). There was no correlation between INR, TT, or aPTT with Kaolin *R* orkaolin Delta, although a moderate correlation (*R* > 0.3, <0.7) was observed between INR, TT, and aPTT with Rapid *R*, Rapid Delta, and Rapid ACT. The Kaolin *α*-Angle did not correlate with fibrinogen (*R* < 0.3), but there was again a moderate correlation between fibrinogen and the Rapid *α*-Angle (Table 4). Excellent correlation (0.84–0.98) was demonstrated between Kaolin TEG and Rapid TEG for the parameters K, MA, G, and LY30 (Table 5).

**Figure 1:**
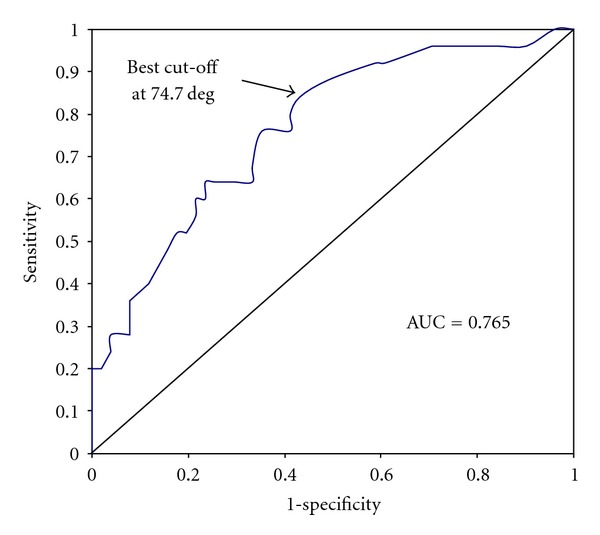
Receiver operator characteristics (ROC) curves for Rapid *α*-Angle predicting transfusion of any blood product.

**Figure 2:**
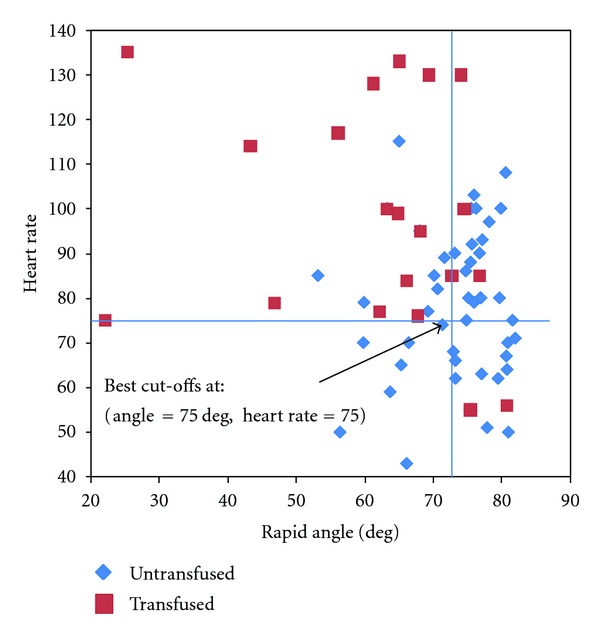
Rapid *α*-Angle plotted against heart rate with calculated cutoff values.

**Figure 3:**
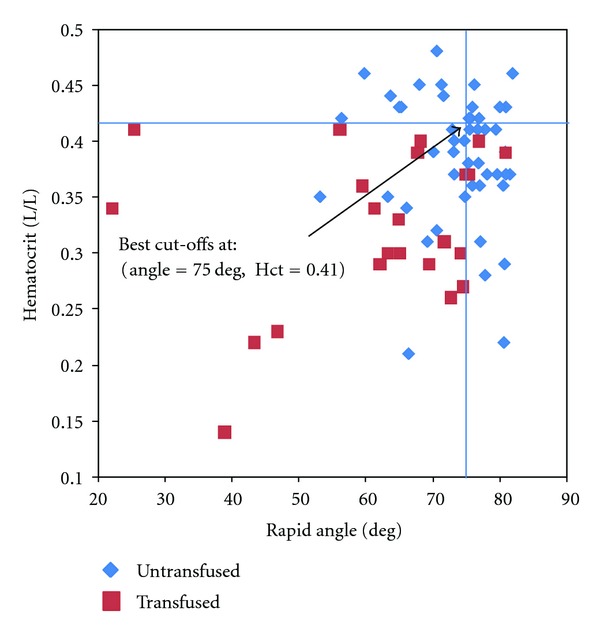
Rapid *α*-Angle plotted against haematocrit with calculated cutoff values.

Sensitivity predicting transfusion was low for all CCT measurements except fibrinogen, although specificity was generally high. The AUC of the receiver operating characteristic (ROC) curves was >70% for all CCT. Kaolin and Rapid TEG were more sensitive than CCT, and the Rapid TEG *α*-Angle was identified as the single parameter with the greatest sensitivity and validity, at a cut-off of 74.7 degrees (Table 6, Figure 1). If the Rapid TEG *α*-Angle is combined with heart rate > 75 bpm or haematocrit <41%, both sensitivity and specificity are improved (Table 6, Figures 2 and 3).

## 4. Discussion

The data of the present study are from a small sample of patients treated in a resuscitation room of a European level 1 trauma centre. This is typical of the European trauma population, where penetrating injuries and exsanguinating hemorrhage are rarer than in US trauma centres. The extent of injury from blunt trauma ranged from minor to serious, although multiple injuries were suspected in all cases on admission to the E.R. Many studies addressing the coagulopathy of trauma focus only on severely injured, massively transfused patients [2, 15]. However, as shown in this study, coagulopathy manifests in a wider range of blunt trauma patients. Overall, 8–28% of patients were characterised by one of the four common definitions of ATC. When the cohort of transfused patients is examined, the incidence of ATC increases to 16–36%. This may be explained by the mechanism of the early coagulopathy of trauma, which is driven by tissue injury, independent of prehospital treatment [16]. Transfusions were appropriately administered to a subset of 25 patients characterised by greater heart rates, more severe injury, and a trend toward higher lactate and greater base deficit. Five patients underwent massive transfusion of ≥10 units of RBCs in 24 hours, and plasma products were concomitantly administered with RBC : FFP ratios approaching 1 : 1.

A major finding of the present study is that there are moderate correlations between individual Rapid TEG parameters and the CCT results. This is also consistent with the literature and our previous study [11, 13]. Additionally, there was excellent correlation between the parameters K, MA, G, and LY30 of Rapid TEG and Kaolin TEG, demonstrating that Rapid TEG can be used in the trauma bay to obtain more rapid results. The mean time to results (Time to MA (TMA)) using Rapid TEG was 18 minutes (Table 3). These data confirm our data from another study, where we were able to show that Rapid TEG results were obtained more rapidly than with Kaolin TEG and conventional coagulation tests [11].

The third finding concerns the predictive ability of CCT and TEG to identify patients who need transfusion. The size of the study is too small to make general recommendations, but the data show that most CCTs lack sensitivity and predictive value. This finding is in accordance with the conclusions of other authors that the current standard of care for coagulation assessment is not a helpful guide for transfusion [4, 8]. This may be due to the long turn around time of lab results, so that decisions on transfusion must be made before the lab results are available [5]. Fibrinogen was the most sensitive CCT, but many trauma centres do not use this test, since results are generally not available for some hours. According to the present data, the Rapid TEG parameter *α*-Angle provided the best sensitivity and validity of all tests examined. If this parameter was used in isolation to predict transfusion, 84% of patients who need transfusion would receive one. Combining the Rapid TEG *α*-Angle (coagulation indicator) with a clinical sign (heart rate) or other point-of-care test (haematocrit) improves the differentiation of patients who require transfusion from those who do not.

In a similar setting, Carroll et al. observed that TEG parameters, especially in combination with PlateletMapping, were more sensitive predictors of blood transfusion and mortality than conventional coagulation tests [17]. However, in our study, positive and negative predictive values were low. This may be related to transfusion decisions which—at the start of trauma resuscitation—are based on clinical parameters and the clinical experience of the trauma leader. Additionally, in the present study, physicians were blinded to TEG results during treatment of trauma patients. Finally, because of the small sample size, cut-off levels for the data set may not be precise enough.

## 5. Conclusion

In this study of moderate to severely injured blunt trauma patients, the results of conventional coagulation tests correlate moderately with Rapid TEG parameters, and Rapid TEG parameters correlate strongly with the respective Kaolin TEG parameters. Since Rapid TEG can provide test results faster than CCT or Kaolin TEG, we suggest further evaluation of Rapid TEG in trauma care, especially in blunt injury. Cut-off points for transfusion can be determined with the Rapid TEG *α*-Angle, which is more sensitive than CCTs. However, a larger study population is needed to reproduce this finding.

## Figures and Tables

**Table 1:** Characteristics of overall study population, nontransfused and transfused patients.

	Overall (*n* = 76)	Nontransfused (*n* = 51)	Transfused (*n* = 25)	*P*
Male (*n*, %)	55 (72)	33 (65)	22 (88)	0.054^a^
Age (years)^c^	49 ± 21	52 ± 20	43 ± 20	0.056^b^
Blunt mechanism (*n*, %)	63 (83)	42 (82)	21 (84)	0.543^b^
Systolic BP (mmHg)	127 ± 23	130 ± 22	122 ± 25	0.111^b^
Diastolic BP (mmHg)	73 ± 19	75 ± 20	70 ± 16	0.399^b^
Heart rate (bpm)	84 ± 21	78 ± 17	98 ± 25	**0.005** ^ b^
Lactate (mmol/L)	2.3 ± 1.8	2.0 ± 1.3	3.0 ± 2.4	0.070^b^
Base deficit (mEq/L)	−2.7 ± 3.3	−1.9 ± 2.1	−3.8 ± 4.4	0.099^b^
Hb (g/dL)	12.4 ± 2.3	13.1 ± 2.0	10.9 ± 2.4	**<0.001**
Hct (%)	36.5 ± 6.8	38.5 ± 5.8	32.3 ± 6.9	**<0.001**
ISS	18 ± 10	15 ± 9	24 ± 10	**0.001** ^ b^
Craniocerebral injury (*n*, %)	33 (43)	20 (39)	13 (52)	0.285^b^
Mortality (*n*, %)	9 (12)	1 (2)	8 (32)	**<0.001** ^ a^
ICU admission (*n*, %)	34 (45)	16 (31)	18 (72)	**0.001** ^ a^
APACHE II score (ICU pts)	19.7 ± 8.3	16.9 ± 8.0	22.3 ± 7.8	0.078^b^
SAPS II	40 ± 19	34 ± 17	45 ± 20	0.101^b^

^a^Fisher's exact test.

^b^Kruskal-Wallis test.

^c^Presented as (mean ± SD) for continuous variables, unless otherwise indicated.

**Table 2:** Patients demonstrating criteria for acute traumatic coagulopathy in overall group, nontransfused and transfused patients.

	Overall	Nontransfused	Transfused	P*
INR > 1.2	14 (18%)	6 (12%)	8 (32%)	**0.012**
INR > 1.5	6 (8%)	2 (4%)	4 (16%)	**0.039**
ATC 1: TT > 15 or aPTT > 60 or INR > 1.2	21 (28%)	12 (24%)	9 (36%)	0.115
ATC 2: TT > 15 or aPTT > 60 or INR > 1.5	17 (22%)	10 (20%)	7 (28%)	0.233

*Kruskal-Wallis test.

**Table 3:** Conventional coagulation test results, Kaolin TEG and rapid TEG measurements in overall group, nontransfused and transfused patients.

	Overall (*n* = 76)	Nontransfused (*n* = 51)	Transfused (*n* = 25)	P*
		CCT		
INR	1.15 ± 0.22	1.10 ± 0.16	1.27 ± 0.29	**0.002**
aPTT (sec)	32 ± 11	30 ± 8	38 ± 13	**0.002**
Thrombin time (sec)	13.1 ± 2.5	12.9 ± 1.4	13.5 ± 3.9	0.689
Fibrinogen (g/L)	2.69 ± 0.98	2.89 ± 0.83	2.23 ± 1.16	**0.002**
Platelet (g/L)	209 ± 57	216 ± 47	194 ± 73	0.331

		Kaolin TEG		
*R* (min)	4.9 ± 1.7	4.9 ± 1.7	5.0 ± 1.7	0.938
*K* (min)	2.1 ± 1.7	1.7 ± 0.5	3.0 ± 2.7	**0.014**
*α*-Angle (deg)	57 ± 12	59 ± 10	53 ± 13	**0.021**
MA (mm)	59 ± 9	62 ± 5	54 ± 12	**0.006**
TMA (min)	24.5 ± 4.0	24.2 ± 3.3	25.0 ± 5.3	0.226
G (d/sec)	7759 ± 2128	8381 ± 1509	6516 ± 2626	**0.006**
LY30 (%)	1.6 ± 10.2	0.5 ± 1.0	3.9 ± 17.7	0.746

		Rapid TEG		
Rapid R (min)	0.85 ± 0.53	0.75 ± 0.39	1.04 ± 0.72	0.106
Rapid Delta (min)	0.20 ± 0.29	0.14 ± 0.13	0.33 ± 0.46	0.119
Rapid ACT	51.80 ± 0.83	51.65 ± 0.60	52.11 ± 1.12	0.106
Rapid K (min)	1.9 ± 1.8	1.4 ± 0.6	3.0 ± 2.6	**<0.001**
Rapid *α*-angle (deg)	70 ± 12	73 ± 7	62 ± 16	**<0.001**
Rapid MA (mm)	59.9 ± 9.0	62.9 ± 5.6	54.0 ± 11.3	**0.001**
Rapid TMA (min)	17.8 ± 4.3	17.0 ± 3.7	19.4 ± 5.0	**0.008**
Rapid G (d/sec)	7933 ± 2458	8659 ± 2032	6480 ± 2625	**0.001**
Rapid LY30 (%)	2.3 ± 10.2	1.1 ± 1.4	4.8 ± 17.6	0.568

*Kruskal-Wallis test.

**Table 4:** Correlation of TEG measurements versus INR, TT, and aPTT.

	Kaolin R	Kaolin Delta	Rapid R	Rapid Delta	Rapid ACT	Kaolin *α*-Angle	Rapid *α*-Angle
INR	0.097	−0.175	0.615	0.476	0.615	—	—
Thrombin time (sec)	0.205	−0.108	0.442	0.505	0.442	—	—
aPTT (sec)	0.275	−0.127	0.561	0.541	0.561	—	—
Fibrinogen	—	—	—	—	—	0.285	0.602

**Table 5:** Correlation of rapid TEG versus Kaolin TEG.

	Correlation coefficient
Rapid ACT versus Kaolin R	0.143
Rapid K versus Kaolin K	**0.936**
Rapid *α*-Angle versus Kaolin *α*-Angle	0.589
Rapid MA versus Kaolin MA	**0.914**
Rapid TMA versus Kaolin TMA	0.517
Rapid G versus Kaolin G	**0.844**
Rapid LY30 versus Kaolin LY30	**0.988**

**Table 6:** Prediction of transfusion by CCT, Rapid TEG*, and Kaolin TEG*.

		Cut-offs	Sensitivity	Specificity	PPV	NPV	AUC
Single indicator							
INR		>1.2	38%	88%	57%	77%	73%
INR		>1.5	19%	96%	67%	74%	73%
aPTT (sec)		>60.0	5%	98%	50%	69%	74%
Fibrinogen (g/L)		<3.0	90%	48%	43%	92%	74%
Thrombin time [sec]		>13.2	48%	73%	45%	75%	53%
Rapid K (min)		>1.8	68%	78%	61%	83%	79%
Kaolin K (min)		>1.7	68%	59%	46%	78%	67%
Rapid *α*-Angle (deg)		**<74.7**	**84%**	**57%**	**49%**	**88%**	**77%**
Kaolin *α*-Angle (deg)		<58.5	72%	61%	47%	82%	66%
Rapid MA (mm)		<59.6	68%	80%	63%	83%	75%
Kaolin MA (mm)		<58.4	56%	88%	70%	80%	70%
Rapid TMA (min)		>17.3	76%	57%	46%	83%	69%
Kaolin TMA (min)		>24.7	64%	63%	46%	78%	58%
Rapid G (d/sc)		<7374	68%	78%	61%	83%	73%
Kaolin G (d/sc)		<7073	56%	88%	70%	80%	70%

Combined indicators							
*α*-Angle + Heart Rate	Rapid *α*-Angle (deg)	<75	**84%**	**75%**	**62%**	**90%**	—
	Heart Rate (bpm)	>75					
*α*-Angle + Hct	Rapid *α*-Angle (deg)	<75	**88%**	**73%**	**61%**	**93%**	—
	Hct (%)	<41					

*Cut-offs determined by the data.
